# Structural characterization of the principal mRNA-export factor Mex67–Mtr2 from *Chaetomium thermophilum*

**DOI:** 10.1107/S2053230X15008766

**Published:** 2015-06-27

**Authors:** Shintaro Aibara, Eugene Valkov, Meindert H. Lamers, Lyudmila Dimitrova, Ed Hurt, Murray Stewart

**Affiliations:** a MRC Laboratory of Molecular Biology, Francis Crick Avenue, Cambridge Biomedical Campus, Cambridge CB2 0QH, England; b Biochemie-Zentrum der Universität Heidelberg, Im Neuenheimer Feld 328, 69120 Heidelberg, Germany

**Keywords:** nuclear transport, Mex67, *Chaetomium thermophilum*, RNA binding

## Abstract

Members of the Mex67–Mtr2/NXF–NXT1 family are the principal mediators of the nuclear export of mRNA. Mex67/NXF1 has a modular structure based on four domains (RRM, LRR, NTF2-like and UBA) that are thought to be present across species, although the level of sequence conservation between organisms, especially in lower eukaryotes, is low. Here, the crystal structures of these domains from the thermophilic fungus *Chaetomium thermophilum* are presented together with small-angle X-ray scattering (SAXS) and *in vitro* RNA-binding data that indicate that, not withstanding the limited sequence conservation between different NXF family members, the molecules retain similar structural and RNA-binding properties. Moreover, the resolution of crystal structures obtained with the *C. thermophilum* domains was often higher than that obtained previously and, when combined with solution and biochemical studies, provided insight into the structural organization, self-association and RNA-binding properties of Mex67–Mtr2 that facilitate mRNA nuclear export.

## Abstract 2

The crystal structures of the individual domains of the Mex67–Mtr2 complex from *C. thermophilum* have been determined and their arrangement in solution has been studied by SAXS.

## 1. Introduction   

**Figure 1:**
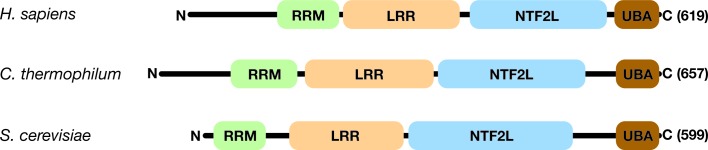
Schematic illustration of the domain structure of Mex67/NXF1 from *H. sapiens*, *C. thermophilum* and *S. cerevisiae*. Although all three organisms retained the four structural domains (RRM, LRR, NTF2L and UBA), *H. sapiens* and *C. thermophilum* had an extended N-terminal region that has been implicated in an auto-regulatory role for NXF1 (Viphakone *et al.*, 2012 ▸).

The Mex67–Mtr2 complex (NXF1–NXT1 in metazoans and *small bristles* in *Drosophila melanogastor*) is the principal mRNA-export factor in *Sacccharomyces cerevisiae* (Segref *et al.*, 1997 ▸; Herold *et al.*, 2000 ▸; Wilkie *et al.*, 2001 ▸). Mex67/NXF1 is a modular protein constructed from four domains (Fig. 1 ▸): an RNA-recognition motif (RRM) domain, a leucine-rich repeat (LRR) domain, a nuclear transport factor 2-like (NTF2L) domain and an ubiquitin-associated (UBA) domain (reviewed by Valkov *et al.*, 2012 ▸). Mtr2 is a ∼15–20 kDa protein that also has an NTF2-like fold and forms a tight heterodimeric complex with the Mex67 NTF2L domain. Mex67–Mtr2 forms direct contacts with mRNA cargoes as well as multiple transient low-affinity interactions with phenyl­alanine–glycine-rich nuclear pore proteins (FG nucleoporins). Translocation of the Mex67–Mtr2+mRNA cargo–carrier complex from the nucleus to the cytoplasm through nuclear pores (NPCs) is thought to rely on rectified Brownian motion, whereby a series of low-affinity interaction with FG nucleoporins enables Mex67–Mtr2 and its associated mRNA cargo to move back and forth within the pore-transport channel, whereas disassembly of the cargo–carrier complex by DEAD-box helicases on the cytoplasmic NPC face is thought to provide the directionality (reviewed by Stewart, 2010 ▸; Valkov *et al.*, 2012 ▸). Classically, binding of the RNA cargo has been attributed to the RRM and LRR domains because together these domains are sufficient for *Homo sapiens* NXF1 to bind the viral RNA CTE (constitutive transport element) sequence motif (Pasquinelli *et al.*, 1997 ▸; Grüter *et al.*, 1998 ▸; Braun *et al.*, 1999 ▸; Wiegand *et al.*, 2002 ▸; Teplova *et al.*, 2011 ▸). Both the NTF2L domain complexed with Mtr2/NXT1 and the UBA domain interact with a range of FG nucleoporins (Fribourg *et al.*, 2001 ▸; Grant *et al.*, 2003 ▸). More recently, the NTF2L domain and Mtr2 have also been implicated in binding ribosomal subunits and rRNA, whereby in *S. cerevisiae* specific long internal loops may be involved in direct RNA binding as well as domain organization to form a continuous RNA-binding platform with the RRM and LRR domains (Yao *et al.*, 2007 ▸; Aibara, Valkov *et al.*, 2015 ▸).

Extensive studies with archaea have demonstrated that proteins obtained from thermophiles often facilitate the determination of high-resolution crystal structures, and this has stimulated interest in eukaryotic thermophiles such as the filamentous fungus *Chaetomium thermophilum* (Bock *et al.*, 2014 ▸). Because the proteins involved in mRNA export tend to have multiple roles and conformational states, often associated with the formation of a series of different complexes during the generation of an export-competent mRNP, high-resolution structures of the components of the pathway are a prerequisite for deciphering the structures of the complexes involved. Moreover, the thermostability of proteins from *C. thermophilum* often facilitates experiments that have proven difficult with their mesophilic counterparts (Leidig *et al.*, 2013 ▸; Monecke *et al.*, 2013 ▸; Thierbach *et al.*, 2013 ▸).

Here, we present the crystal structures of the individual domains of *C. thermophilum* Mex67–Mtr2 (*ct*Mex67–Mtr2) to provide a complete repertoire of high-resolution structures from a single species to facilitate structural studies of the complexes formed during the formation of export-competent mRNPs. *In vitro* SAXS and RNA-binding studies have also demonstrated that *ct*Mex67–Mtr2 has similar biochemical properties as *H. sapiens* NXF1–NXT1 (*hs*NXF1–NXT1) and, in particular, have shown that the ctMex67 NTF2L domain contributes to mRNA binding.

## 2. Methods   

### 2.1. Cloning and protein purification   

Genes corresponding to *ct*Mex67 and *ct*Mtr2 were PCR-amplified from *C. thermophilum* cDNA using standard procedures. Fragments corresponding to the RRM (*ct*Mex67^RRM^; residues 93–200) and LRR (*ct*Mex67^LRR^; residues 191–360) domains were cloned into the first multiple cloning site (MCS) of pETDuet-1 to generate His_6_-tagged constructs. Fragments corresponding to the NTF2L domain (*ct*Mex67^NTF2L^; residues 365–564) and *ct*Mtr2 were cloned into the first and second MCS sites of pETDuet-1, respectively, to generate His_6_-tagged *ct*Mex67^NTF2L^–Mtr2. A fragment corresponding to the UBA domain (*ct*Mex67^UBA^; residues 600–657) was cloned into pMCSG10 to generate a TEV-cleavable GST-tagged construct. Longer constructs containing multiple domains of *ct*Mex67 [LRR-NTF2L (residues 180–556), ΔNΔUBA (residues 93–556) and ΔN (residues 70–657)] and Mtr2 were also cloned into the first and second MCS sites of pETDuet-1, respectively, to generate TEV-cleavable His_6_-tagged constructs. All protein constructs were expressed in *Escherichia coli* BL21-CodonPlus(DE3)-RIL cells by IPTG induction at 291 K over 16 h and all purification steps and manipulations were conducted at 277 K unless stated otherwise. Harvested cell pellets were resuspended at 5 ml per gram of wet cell pellet in 50 m*M* Tris–HCl pH 7.4, 500 m*M* NaCl, 20 m*M* imidazole pH 8.0 (His_6_-tagged constructs) or 50 m*M* Tris–HCl pH 7.4, 500 m*M* NaCl, 5 m*M* DTT (GST-tagged constructs) and lysed by high-pressure cavitation using two passes through an EmulsiFlex C3 (Avestin). Lysates were clarified by centrifugation and the supernatant was incubated with Ni–NTA agarose beads or glutathione Sepharose 4B beads for 1 h. Nonspecifically bound proteins were removed by washing with the buffer used for lysis, after which the His_6_-tagged proteins were eluted with 50 m*M* Tris–HCl pH 7.4, 500 m*M* NaCl, 250 m*M* imidazole and GST-tagged proteins were eluted with 50 m*M* Tris–HCl pH 7.4, 500 m*M* NaCl, 5 m*M* DTT, 2 m*M* reduced glutathione. TEV-cleavable tags were then removed by incubation with TEV protease overnight. Constructs with more than one domain were purified further using heparin affinity chromatography as described in Aibara, Valkov *et al.* (2015 ▸). All proteins were finally purified to homogeneity by size-exclusion chromatography using a HiLoad 26/60 Superdex 75 or 200 column equilibrated in 20 m*M* Na HEPES pH 8.0, 200 m*M* NaCl (for all multi-domain constructs), 20 m*M* Na HEPES pH 8.0, 750 m*M* NaCl, 5 m*M* DTT (for *ct*Mex67^UBA^) or 20 m*M* Tris–HCl pH 7.4, 50 m*M* NaCl (for all other single-domain constructs). Small aliquots of purified proteins were flash-frozen in liquid nitrogen and stored at 193 K until required.

### 2.2. X-ray crystallography   

Protein crystals were grown at 291 K by sitting-drop vapour diffusion in which 200 nl purified protein solution was mixed with 200 nl well buffer (see Table 1 ▸) and cryocooled in mother liquor supplemented with 20% glycerol. X-ray diffraction data were collected on beamlines I04, I04-1 and I24 at Diamond Light Source, Didcot, England. Reflections were indexed and integrated using *XDS* (Kabsch, 2010 ▸) and then scaled and merged in *AIMLESS*, ensuring a completeness of >98% in the outermost shell while maintaining CC_1/2_ > 0.3 (Evans & Murshudov, 2013 ▸). The *ct*Mex67^RRM^, *ct*Mex67^LRR^ and *ct*Mtr2 structures were obtained by molecular replacement using the *MR-Rosetta* pipeline (DiMaio *et al.*, 2011 ▸) with human RRM and LRR domains (Teplova *et al.*, 2011 ▸; PDB entry 3rw7) and *sc*Mtr2 (Fribourg & Conti, 2003 ▸; PDB entry 1of5) as search models, whereas *ct*Mex67^NTF2L^–Mtr2 was solved by molecular replacement using *Phaser* (McCoy *et al.*, 2007 ▸) with the *S. cerevisiae* homologue (Fribourg & Conti, 2003 ▸; PDB entry 1of5) and *ct*Mtr2 (PDB entry 4x2m) as search models. The structure of *ct*Mex67^UBA^ was solved by SAD phasing in *Phaser* (McCoy *et al.*, 2007 ▸) using anomalous scattering from *S*-(dimethylarsenic)cysteine that was derived from cysteines modified by the cacodylate buffer (Maignan *et al.*, 1998 ▸; Liu *et al.*, 2011 ▸). Iterative cycles of rebuilding using *Coot* (Emsley *et al.*, 2010 ▸) and refinement using *PHENIX* (Adams *et al.*, 2010 ▸) with weightings chosen to minimize *R*_free_ were used to generate the final models (Table 1 ▸).

### 2.3. Small-angle X-ray scattering   

Small-angle X-ray scattering (SAXS) data were collected on beamline BM29 at the European Synchrotron Radiation Facility, Grenoble, France using an online HPLC system (Viscotek) equipped with a Superdex 200 Increase 3.2/300 column (GE Healthcare) with a sample flow rate of 0.1 ml min^−1^. Data were collected at 293 K using a wavelength of 0.995 Å and a sample-to-detector distance of 1 m, and were processed automatically using the online *AUTOSUB* pipeline (Konarev *et al.*, 2003 ▸). All gave linear Guinier plots for s*R_g_ < 1.3. Pair distance distribution functions of the particles *P*(*r*) and their maximum sizes *D*_max_ were computed using *GNOM* (Svergun, 1992 ▸) and molecular weights were estimated by comparing the extrapolated forward scattering of the samples obtained from Guinier analysis using *AUTORG* (Konarev *et al.*, 2003 ▸) with that of a bovine serum albumin standard (Sigma–Aldrich). Theoretical SAXS profiles based on atomic models and comparisons with experimental SAXS profiles were obtained using the *FoXS* server (Schneidman-Duhovny *et al.*, 2010 ▸, 2013 ▸).

### 2.4. *In vitro* RNA-binding assays   

The interaction of RNA with a range of *ct*Mex67 domain-based constructs was assessed using fluorescence anisotropy, in which 10 n*M* DY-547-labelled homopolymeric RNA (polyA_15_, polyU_15_, polyC_15_, polyG_15_) was mixed with serially diluted protein in 20 m*M* Tris–HCl pH 8.0, 50 m*M* NaCl as described previously (Aibara, Valkov *et al.*, 2015 ▸).

## 3. Results and discussion   

### 3.1. Structural conservation of domains in *C. thermophilum* Mex67   

Although *ct*Mex67 shared only 23.0% sequence identity with *sc*Mex67 and 23.5% identity with *hs*NXF1, the crystal structures obtained for the RRM domain (2.4 Å resolution), the LRR domain (1.7 Å resolution), *ct*Mtr2 alone (2.0 Å resolution), NTF2L–Mtr2 (2.9 Å resolution) and the UBA domain (1.7 Å resolution) of *ct*Mex67–Mtr2 (Table 1 ▸) generally retained the key features of their yeast and metazoan counterparts, albeit with several differences.

**Figure 2:**
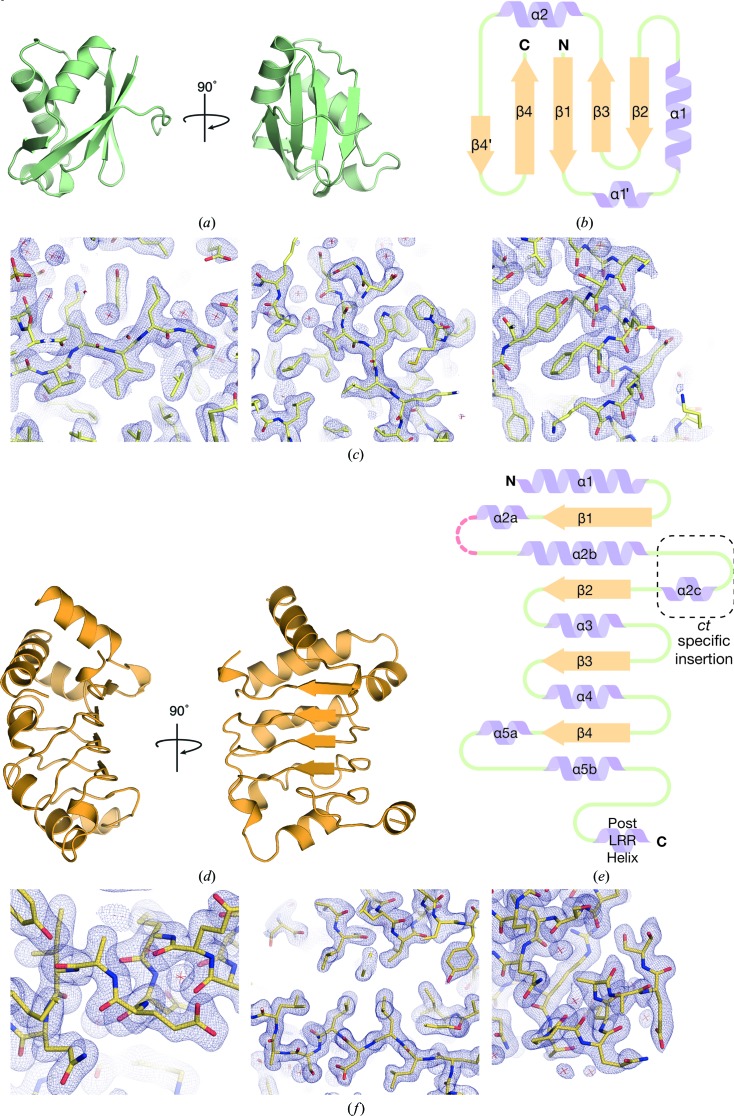
(*a*) Overview of the 2.4 Å resolution crystal structure of *ct*Mex67^RRM^. (*b*) A schematic illustration of the secondary-structural elements present in the RRM domain, which showed the characteristic βαββαβ fold. The RRM domain from *ct*Mex67 had a short β-strand prior to β4 that was not found in other organisms (denoted β4′). (*c*) Three representative views of the final 2*F*_o_ − *F*_c_ maps for the *ct*Mex67^RRM^ structure contoured at the 1σ level. (*d*) Overview of the 1.7 Å resolution crystal structure of *ct*Mex67^LRR^. (*e*) Schematic illustration of the secondary-structural elements present in the LRR domain whereby tandem repeating α-helices and β-sheets generate a curved structure. Disordered regions are shown as red dotted lines. The LRR domain from *ct*Mex67 includes an extra helix insertion between α2b and β2 when compared with the *H. sapiens* homologue. (*f*) Three representative views of the final 2*F*_o_ − *F*_c_ maps for the *ct*Mex67^LRR^ structure contoured at the 1σ level.

The 2.4 Å resolution crystal structure of *ct*Mex67^RRM^ resembled that of *hs*NXF1^RRM^ (Liker *et al.*, 2000 ▸; Teplova *et al.*, 2011 ▸; PDB entries 1fo1 and 3rw6) and was based on the characteristic RRM βαββαβ fold (Figs. 2 ▸ *a*, 2 ▸ *b* and 2 ▸ *c*). Like other NXF family members, *ct*Mex67^RRM^ did not contain a typical RRM consensus sequence that is based on two motifs: RNP1, (K/R)_o_-G-(F/Y)_o_-(G/A)_i_-F_o_-V_i_-X_o_-(F/Y)_i_, and RNP2, (L/I)_i_-(Y/F)_o_-(V/I)_i_-(G/N)_o_-(G/N)_o_-(L/M)_i_, where the subscript ‘o’ or ‘i’ signifies whether the side chain is surface-exposed or facing the core of the RRM fold (Liker *et al.*, 2000 ▸; Maris *et al.*, 2005 ▸). In *ct*Mex67^RRM^ the RNP1 (residues 143–150) and RNP2 (residues 101–106) sequences were G-D_o_-Y_o_-V_i_-W_o_-L_i_-K_o_-V_i_ and I_i_-K_o_-I_i_-L_o_-G-L_i_, respectively. The RNP2 motif appeared to be more conserved than RNP1, although the characteristic aromatic group at position 2 that typically forms ring-stacking interactions with RNA was instead lysine in *ct*Mex67^RRM^. The large deviations from the RNP1 and RNP2 sequences found in different members of the NXF1/Mex67 family may reflect that they bind RNA in a more general, non-base-specific way, as appeared to be the case in the crystal structure of *hs*NXF1^RRM-LRR^–CTE-B, in which the *hs*NXF1 RRM domain formed mainly phosphate–backbone interactions with the CTE-B RNA (Teplova *et al.*, 2011 ▸). The 1.7 Å resolution crystal structure of *ct*Mex67^LRR^ showed the characteristic pattern of alternating α-helices and β-strands arranged to form a gently curving structure similar to that seen in this domain in homologous structures (Figs. 2 ▸ *d*, 2 ▸ *e* and 2 ▸ *f*; Liker *et al.*, 2000 ▸; Teplova *et al.*, 2011 ▸), although *ct*Mex67^LRR^ had an additional α-helix inserted between the first α-helix and β-strand of the domain core (Fig. 2 ▸ *e*; denoted α2c).

**Figure 3:**
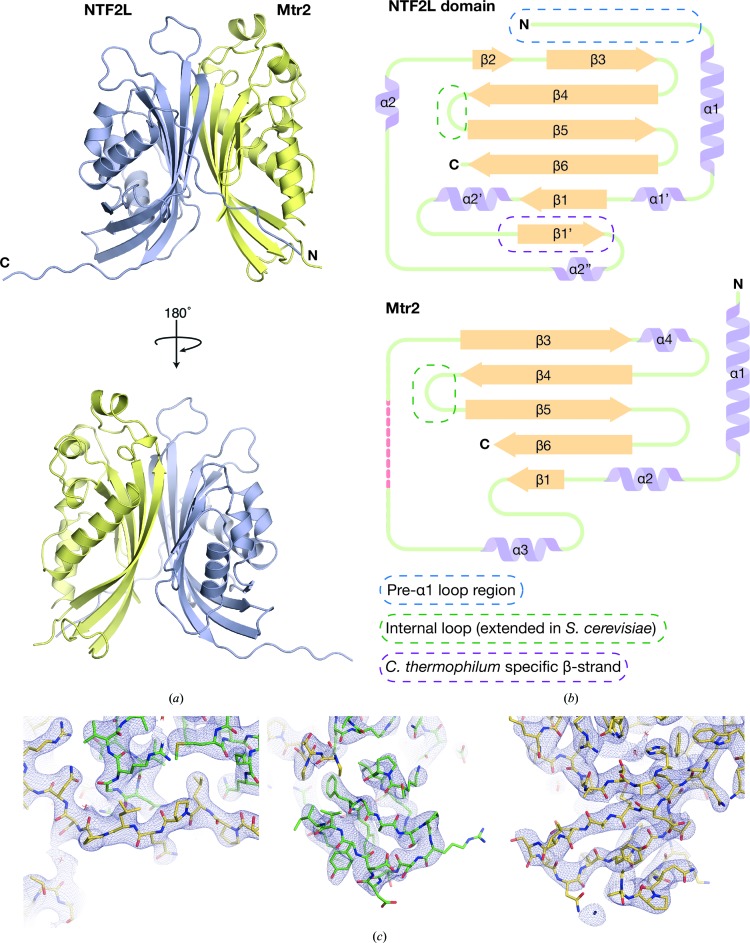
(*a*) Overview of the 2.9 Å resolution crystal structure of *ct*Mex67^NTF2L^–Mtr2. The two chains are related in a twofold-symmetric manner, where the highly curved β-sheets form a tight heterodimeric complex. (*b*) Schematic illustration of the secondary-structural elements in the NTF2L domain and Mtr2. Disordered regions are shown as red dotted lines. The internal loop present between β4 and β5 in both the NTF2L domain and Mtr2 were ordered, but not extended as shown to be the case in *S. cerevisiae* (circled with a dotted green line). The pre-α1 loop region of the NTF2L domain was also ordered in *ct*Mex67 and was bound across Mtr2 in an analogous way to that seen in *hs*NXF1^NTF2L^–NXT1 (PDB entry 1jkg; Fribourg *et al.*, 2001 ▸). An extra β-­strand was present in the NTF2L domain when compared with the *hs*NXF1 NTF2L domain and was probably owing to a lattice contact involving the extreme C-terminus of the NTF2L domain (denoted β1′ and circled with a dotted purple line). (*c*) Three representative views of the final 2*F*_o_ − *F*_c_ maps for the *ct*Mex67^NTF2L^–Mtr2 structure contoured at the 1σ level (the *ct*Mex67^NTF2L^ domain is shown in yellow and *ct*Mtr2 is shown in green).

**Figure 4:**
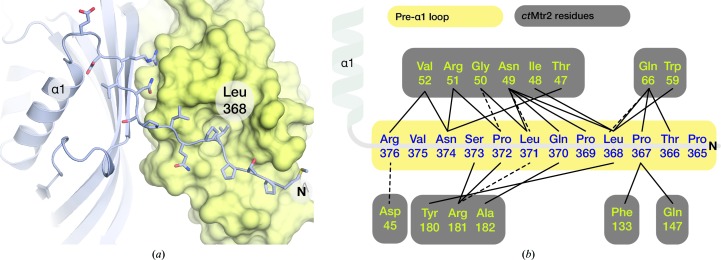
(*a*) Detailed view of the pre-α1 loop region (represented as sticks) spanning the surface of Mtr2 (yellow). Hydrophobic contacts between Mex67 and Mtr2 centring on Leu368 of Mex67 were found outside the NTF2-like core. (*b*) Schematic representation of the interactions between the pre-α1 loop (yellow) and Mtr2 (grey) found outside the NTF2-like core. Solid lines represent hydrophobic interactions and dotted lines represent putative hydrogen bonds.

The *ct*Mex67^NTF2L^ and *ct*Mtr2 chains interacted through their highly curved β-sheets in a pseudo-twofold-symmetric manner in the 2.9 Å resolution crystal structure, similar to that observed in the analogous complexes from *H. sapiens* and *S. cerevisiae* (Fribourg *et al.*, 2001 ▸; Fribourg & Conti, 2003 ▸). In the complex, *ct*Mtr2 was not altered when compared with the isolated structure, with a C^α^ r.m.s.d. of 0.38 Å over 148 residues. Although the folds of the NTF2-like domains in both Mex67 and Mtr2 were similar across species, those in *ct*Mex67^NTF2L^–Mtr2 had more extensive and longer loops and β-sheets than those observed in *H. sapiens* and *S. cerevisiae* (Fig. 3 ▸ *a*). Neither *ct*Mex67^NTF2L^ nor *ct*Mtr2 had the larger internal loops between strands β4 and β5 present in *S. cerevisiae* but which were disordered in previous structures (Fribourg & Conti, 2003 ▸; Fig. 3 ▸ *b*). These loops have been implicated in pre-60S ribosomal export, where a surface flanked by these loops is formed on the NTF2L–Mtr2 region that is separate from that used for bulk mRNA export (Yao *et al.*, 2007 ▸, 2008 ▸) and also contribute to the ordered arrangement of domains in *S. cerevisiae* Mex67 (Aibara, Valkov *et al.*, 2015 ▸). There was clear density for these loops in the *ct*Mex67^NTF2L^–Mtr2 crystal structure, but as in *hs*NXF1^NTF2L^–NXT1 they did not form an extended surface, suggesting that *ct*Mex67–Mtr2 may not be involved in ribosomal export in *C. thermophilum* in the quite same way as in *S. cerevisiae*. A crystal contact in which the C-terminal region (residues 557–564) of the *ct*Mex67^NTF2L^ chain made contact with the cavity formed between α2′ and β1′ from a symmetry-related *ct*Mex67^NTF2L^ chain resulted in *ct*Mex67^NTF2L^ having an additional β-strand (β1′ in Fig. 3 ▸ *b*) compared with *H. sapiens* NXF1^NTF2L^, although the biological significance of this interaction remains to be established. There was clear density for the N-terminal region of *ct*Mex67^NTF2L^ (the pre-α1 loop, residues 365–376) that placed this region in a position in which it spanned across the surface of *ct*Mtr2 (Fig. 4 ▸ *a*). In particular, Leu368 was buried in a hydrophobic pocket present in *ct*Mtr2. This arrangement of the Mex67^NTF2L^ pre-α1 loop is also present in the *H. sapiens* homologue as well as in the multi-domain structure of *S. cerevisiae* Mex67^ΔUBA^–Mtr2 (Fribourg *et al.*, 2001 ▸; Aibara, Valkov *et al.*, 2015 ▸). In *S. cerevisiae*, the pre-α1 loop formed a rich network of interactions that appeared to make a major contribution to the spatial arrangement of Mex67 domains (Fig. 4 ▸ *b*) and thus the observation that this interaction is conserved between *S. cerevisiae*, *C. thermophilum* and *H. sapiens* indicates that it represents a conserved structural feature within these complexes.

**Figure 5:**
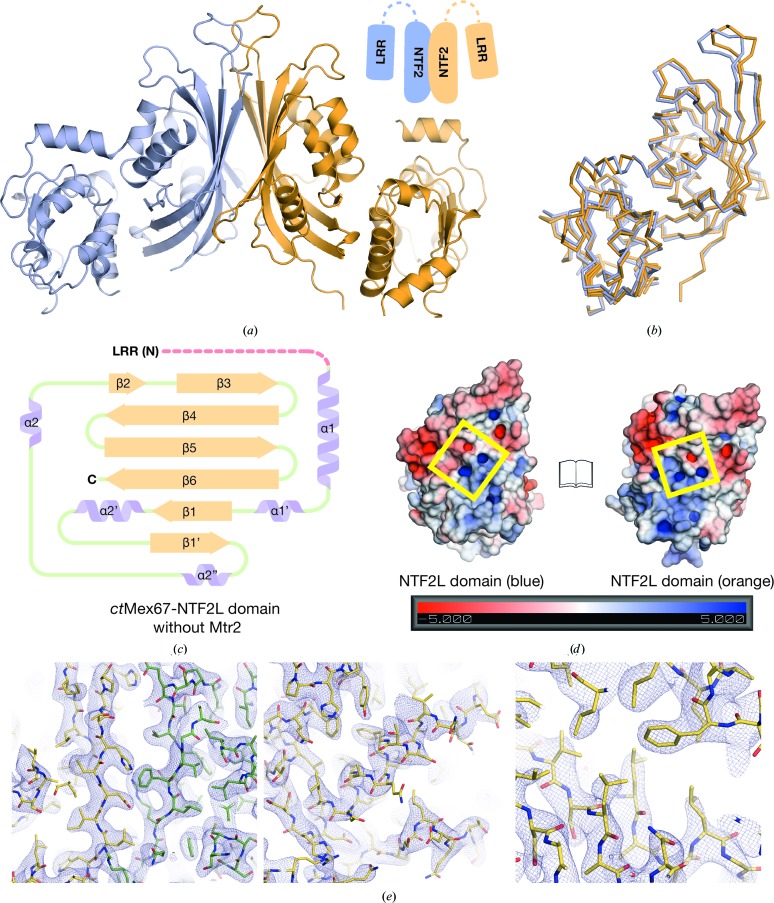
(*a*) Overview of the 2.95 Å resolution crystal structure of *ct*Mex67^LRR-NTF2L^. Two copies of the protein in the asymmetric unit were assumed in a homodimeric configuration analogous to that of *S. cerevisiae* NTF2 (Bayliss *et al.*, 2002 ▸; PDB entry 1gyb). Residues corresponding to the LRR-NTF2L linker (residues 362–378) were disordered as depicted in the schematic representation using dotted lines. (*b*) Structural alignment of the two copies of *ct*Mex67^LRR-NTF2L^ in the asymmetric unit; the LRR domain was placed in the same position with respect to the NTF2L domain in both copies. A C^α^ r.m.s.d. of 1.03 Å was observed over 294 residues. (*c*) Schematic of the secondary-structure elements present in the NTF2L domain for the structure of *ct*Mex67^LRR-NTF2L^. No major changes in the NTF2-like core were observed, although rearrangements in the loop regions were detected. The pre-α1 loop which was previously ordered in the structure of *ct*Mex67^NTF2L^–Mtr2 was disordered in this structure (depicted as a dotted red line). (*d*) View of the electrostatic surface potential of the β-sheet interface between the two NTF2L domains. The ‘NXF plug’ previously identified to confer specificity for the Mex67–Mtr2 interaction (Kerkow *et al.*, 2012 ▸) was still present in this structure of homodimeric Mex67. (*e*) Three representative views of the final 2*F*_o_ − *F*_c_ maps for the *ct*Mex67^LRR-NTF2L^ structure contoured at the 1σ level (one copy of *ct*Mex67^LRR-NTF2L^ is shown in yellow and the other is shown in green).

The 2.95 Å resolution *ct*Mex67^LRR-NTF2L^ crystal structure had two copies of the protein in the asymmetric unit that generated a pseudo-homodimer through extensive inter­actions between the highly curved β-sheets of each Mex67 NTF2L domain (Fig. 5 ▸ *a*) that were analogous to those seen in the *S. cerevisiae* Mex67–Mtr2 heterodimer or NTF2 homodimer (Bayliss *et al.*, 2002 ▸). Two copies of the LRR domain were also present in the asymmetric unit, but the LRR-NTF2L linker was disordered. However, both LRR domains had the same orientation with respect to *ct*Mex67^NTF2L^, and when aligned the two copies had a C^α^ r.m.s.d. of 1.03 Å (Fig. 5 ▸ *b*). Comparison of the fold of *ct*Mex67^NTF2L^ in this structure with that observed when it was complexed with Mtr2 indicated that the major secondary-structural elements of the NTF2-like core were not perturbed, although there were several rearrangements in the loop regions (Fig. 5 ▸ *c*). However, the previously ordered pre-α1 loop that gave rise to the asymmetry in the NTF2L–Mtr2 region was now disordered, perhaps thereby allowing the symmetric homodimeric Mex67 to be generated. Although it has been proposed that a motif known as the ‘NXF plug’ formed by residues present in the core of the NTF2L domain would inhibit Mex67 homodimerization (Kerkow *et al.*, 2012 ▸), this motif was still present in the current structure (Fig. 5 ▸ *d*). Moreover, *ct*Mex67^LRR-NTF2L^ appeared to form a homodimer in solution, and in size-exclusion chromatography coupled with multi-angle light scattering the protein migrated as a single peak with a molecular mass of 82 g mol^−1^ (Supplementary Fig. S2), consistent with a dimer (each chain has a theoretical mass of 42 g mol^−1^).

**Figure 6:**
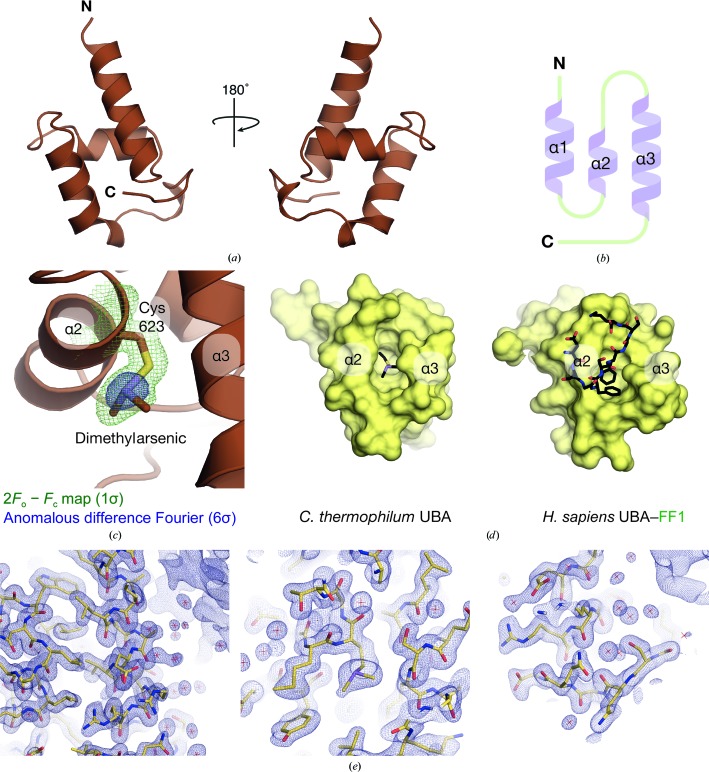
(*a*) Overview of the 1.7 Å resolution crystal structure of *ct*Mex67^UBA^. Like other UBA domains of Mex67/NXF1 from *H. sapiens* and *S. cerevisiae*, the domain was based on three principal α-helices together with the extreme C-terminal region that formed contacts with the first α-helix (α1). (*b*) Schematic illustration of the secondary-structural elements in the UBA domain. (*c*) Detailed view of the dimethylarsenic group conjugated to Cys623 of *ct*Mex67. An anomalous difference Fourier contoured at 6σ (represented in blue) showed clear density around the As atom. (*d*) Surface representation of the FG nucleoporin binding site present in the UBA domains of *ct*Mex67 (left) and *hs*NXF1 (right). The UBA domain from *ct*Mex67 clearly has the same binding pocket as present in *hs*NXF1, although the dimethylarsenic group described in (*c*) was found to be bound there. (*e*) Three representative views of the final 2*F*_o_ − *F*_c_ map for the *ct*Mex67^UBA^ structure contoured at the 1σ level.

The 1.7 Å resolution crystal structure of *ct*Mex67^UBA^ showed a clear As anomalous signal from a dimethylarsenic group on Cys623 that was derived from the cacodylate buffer (Figs. 6 ▸ *a*, 6 ▸ *b* and 6 ▸ *c*). The *ct*Mex67^UBA^ fold was based on three α-helices similar to those seen in *H. sapiens* and *S. cerevisiae* (Grant *et al.*, 2003 ▸; Hobeika *et al.*, 2009 ▸) and there was a hydrophobic pocket in an equivalent position to where a FG nucleoporin peptide binds in the *H. sapiens* homologue (Grant *et al.*, 2003 ▸), indicating that *ct*Mex67^UBA^ probably also interacts with FG nucleoporins in a similar fashion. Cys623 was also located in this solvent-accessible pocket (Fig. 6 ▸ *d*). The *ct*Mex67 UBA domain contained a four-residue C-terminal extension compared with the *H. sapiens* homologue that formed interactions with the end of α1 in seven copies of the eight in the asymmetric unit and which might contribute to thermostability.

In summary, the fold of all four domains of *C. thermophilum* Mex67 and Mtr2 was conserved when compared with the *H. sapiens* homologue and displayed a low C^α^ r.m.s.d. values when aligned structurally across species (Table 2 ▸). The UBA domains and Mtr2/NXT1 showed a high level of structural conservation (C^α^ r.m.s.d. < 0.8 Å), whereas the NTF2L domain showed the greatest variation (C^α^ r.m.s.d. = 1.9 Å). However, despite the conservation of the fold, even after structure-based alignment the number of identical equivalent residues remained remarkably low. The UBA domain showed the greatest conservation (29% sequence identity over 55 residues), whereas the RRM domain had the lowest (15% identity over 73 residues). An unexpected homodimeric form of *ct*Mex67 was also observed in which the two copies of *ct*Mex67^NTF2L^ formed a dimer analogous to that in *S. cerevisiae* NTF2.

### 3.2. SAXS indicates that *C. thermophilum* and *S. cerevisiae* Mex67–Mtr2 have a similar spatial arrangement of domains   

**Figure 7:**
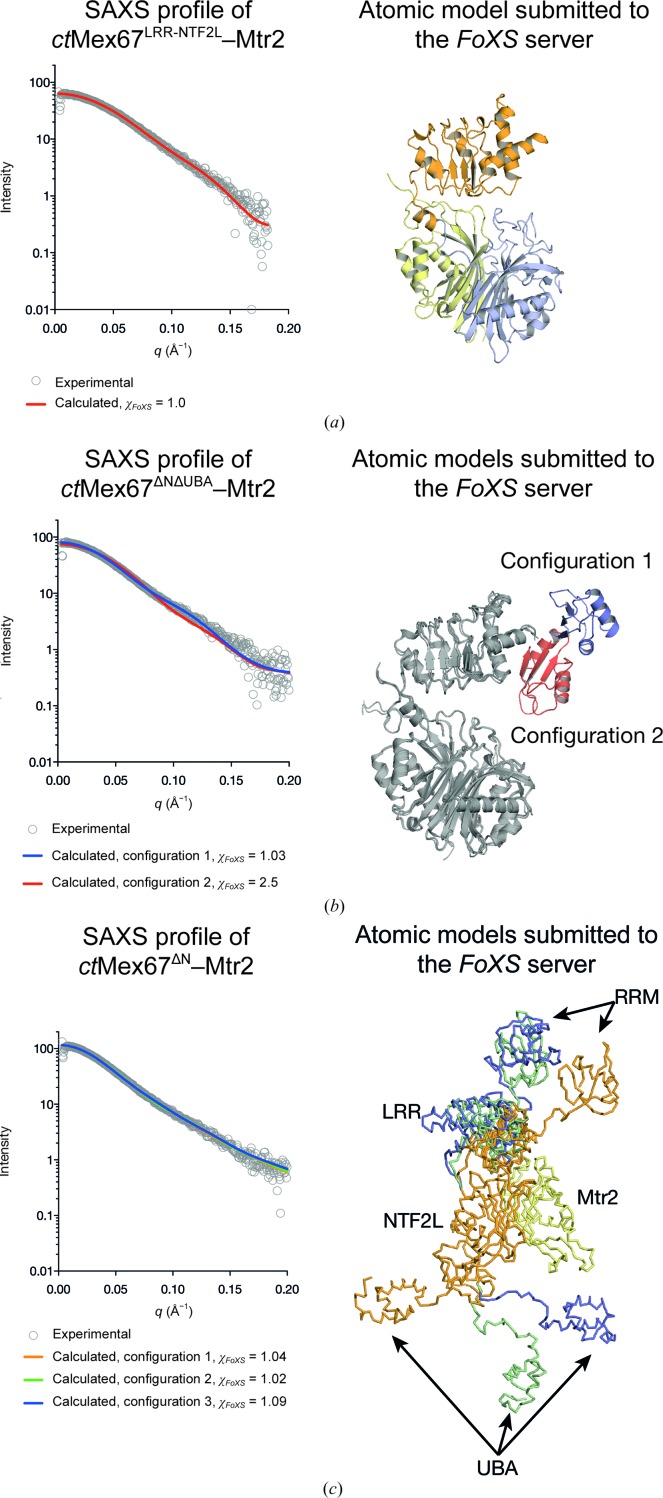
(*a*) The theoretical scattering of *sc*Mex67^LRR-NTF2L^–Mtr2 matched the observed SAXS profile for the equivalent *ct*Mex67–Mtr2 construct. (*b*) The theoretical scattering of the two configurations of the RRM domain present in the crystal structure of *sc*Mex67^ΔUBA^–Mtr2 fitted to varying degrees: configuration 1 (blue) fitted the SAX data well, whereas configuration 2 (red) fitted less well. (*c*) A range of atomic models of the *ct*Mex67^ΔN^–Mtr2 were generated by *BILBOMD* and the three best models had excellent fits to the experimental SAXS profile. The spatial arrangement of the LRR domain relative to the NTF2L domain was conserved between models, whereas the position of the UBA domain was variable.

Previous studies indicated that there is a defined spatial relationship between the Mex67 RRM, LRR and NTF2L domains (Aibara, Valkov *et al.*, 2015 ▸). Despite extensive crystallization trials, crystals of constructs containing multiple domains of *ct*Mex67 complexed with *ct*Mtr2 could not be obtained, and therefore small-angle X-ray scattering (SAXS) was used to investigate the solution state of *ct*Mex67–Mtr2 using systematic truncations of domains (Table 3 ▸). Because the *sc*Mex67–Mtr2 RRM-LRR linker showed some flexibility (Aibara, Valkov *et al.*, 2015 ▸), a *ct*Mex67^LRR-NTF2L^–Mtr2 complex was initially examined. The theoretical SAXS profile generated from an atomic model of *sc*Mex67^LRR-NTF2L^–Mtr2 showed an excellent fit to the *C. thermophilum* data (χ_FoXS_ = 1.0), consistent with this domain arrangement probably being conserved. The *sc*Mex67^ΔUBA^–Mtr2 crystal structure (Aibara, Valkov *et al.*, 2015 ▸) showed two different RRM-LRR domain arrangements that were tested separately, but whereas configuration 1 (Fig. 7 ▸ *b*, blue) fitted the SAXS profile of *ct*Mex67^ΔNΔUBA^–Mtr2 well (χ_FoXS_ = 1.03), configuration 2 (Fig. 7 ▸ *b*, red) fitted less well (χ_FoXS_ = 2.5). Models of *ct*Mex67^ΔN^–Mtr2 were generated by molecular dynamics in *BILBOMD* (Pelikan *et al.*, 2009 ▸) to investigate the position of the UBA domain relative to the rest of Mex67–Mtr2. The individual Mex67 domains were joined by polyalanine linkers and then allowed to move as rigid bodies while scoring the fit against the measured SAXS profile of *ct*Mex67^ΔN^–Mtr2. The three best models all had excellent fits [χ_FoXS_ = 1.04, 1.02 and 1.09 for configurations 1 (orange), 2 (green) and 3 (blue), respectively; Fig. 7 ▸ *c*]. Aligning the three best models based on the NTF2L–Mtr2 region showed that consistent with the SAXS data obtained with Mex67^LRR-NTF2L^–Mtr2, the RRM domain showed flexibility whereas the LRR domain position remained constant. Strikingly, the UBA domain in these models was placed in three very different positions, consistent with the spatial positions of the NTF2L and UBA domains not being strongly constrained.

### 3.3. RNA binding of *ct*Mex67–Mtr2   

**Figure 8:**
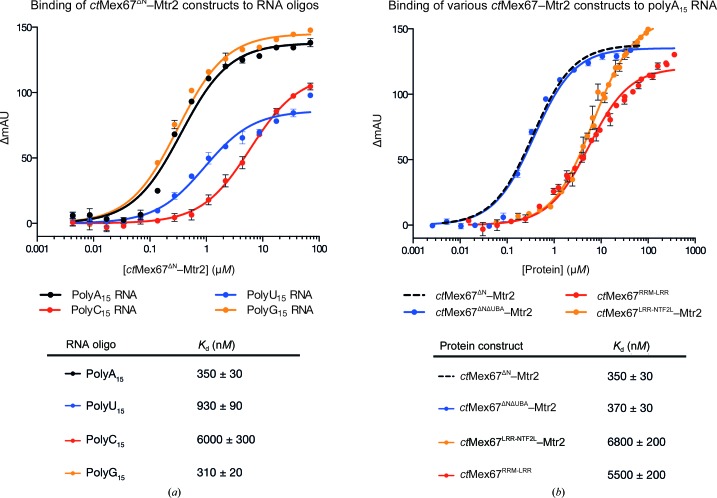
(*a*) Fluorescence anisotropy assays using *ct*Mex67^ΔN^–Mtr2. Data were fitted to the standard quadratic binding equation to obtain *K*_d_ values. All four RNA oligonucleotides tested were bound, albeit with different affinities. Similar to *sc*Mex67–Mtr2, *ct*Mex67^ΔN^–Mtr2 bound polyA_15_ and polyG_15_ more tightly than polyU_15_ and polyG_15_. (*b*) Deletion of either the RRM or NTF2L–Mtr2 domains reduced the affinity of *ct*Mex67 by >15-fold. On the other hand, deletion of the UBA domain from *ct*Mex67^ΔN^–Mtr2 did not reduce the affinity towards polyA_15_ RNA significantly. Curves reproduced from (*a*) are shown as dotted lines without data points.

Fluorescence anisotropy assays (Fig. 8 ▸) showed that *ct*Mex67–Mtr2 bound all four RNA oligonucleotides tested (A_15_/U_15_/C_15_/G_15_) *in vitro* but with different affinities. Similar to that from *S. cerevisiae*, *ct*Mex67–Mtr2 bound polyA_15_ and polyG_15_ (*K*_d_ of 350 and 310 n*M*, respectively) more strongly than polyU_15_ and polyC_15_ (*K*_d_ of 930 and 6.0 µ*M*, respectively). Removal of the UBA domain did not reduce the affinity significantly and the observed *K*_d_ for *ct*Mex67^ΔUBA^–Mtr2 (370 n*M*) was indistinguishable from that for the complete complex (350 n*M*). However, deletion of either the RRM domain or the NTF2L–Mtr2 region reduced the affinity more than 15-fold. Thus, the *K*_d_ for the *ct*Mex67^LRR-NTF2L^–Mtr2 (RRM domain deletion) was 6.8 µ*M*, whereas that for *ct*Mex67^RRM-LRR^ (NTF2L–Mtr2 region deletion) was 5.5 µ*M*. These data indicated that as in *S. cerevisiae* (Aibara, Valkov *et al.*, 2015 ▸) the RRM, LRR and NTF2L–Mtr2 regions all contribute to RNA binding by *ct*Mex67–Mtr2. Indeed, the RRM domain and the NTF2L–Mtr2 region appeared to contribute roughly equally, since removal of either reduced the affinity for polyA_15_ RNA by ∼15-fold. This result contrasts with *sc*Mex67–Mtr2, where removal of the RRM domain had a smaller impact on the binding than removal of the NTF2L–Mtr2 region (Aibara, Valkov *et al.*, 2015 ▸). The apparent differences in the contributions of the RRM and NTF2L domains may reflect differences between organisms, since Liker *et al.* (2000 ▸) showed that although *hs*NXF1^RRM-LRR^ bound RNA *in vitro* the equivalent *sc*Mex67 construct did not. Similarly, the NTF2L–Mtr2 region of *sc*Mex67–Mtr2 has been proposed to have extended loops that contribute to binding ribosomal RNA (Yao *et al.*, 2008 ▸) but which are absent in *hs*NXF1–NXT1. The observation that *ct*Mex67^RRM-LRR^ showed a stronger affinity for RNA than that observed with *sc*Mex67^RRM-LRR^, whereas the NTF2L–Mtr2 region appeared to make a smaller contribution, suggests that from the perspective of RNA binding *ct*Mex67–Mtr2 may be more similar to *hs*NXF1–NXT1. The structure of *sc*Mex67^ΔUBA^–Mtr2 (Aibara, Valkov *et al.*, 2015 ▸; PDB entry 4wwu) indicated that a continuous RNA-binding interface could be generated from the way in which the LRR and NTF2L domains of Mex67 assumed a defined three-dimensional arrangement through interactions with Mtr2. Although this arrangement has not directly been observed for *ct*Mex67–Mtr2 using X-ray crystallography, binding assays identifying interactions between RNA and the NTF2L–Mtr2 region in addition to the RRM and LRR domains are consistent with *ct*Mex67–Mtr2 adopting a similar conformation to generate an extended positively charged region to mediate RNA binding.

In summary, the crystal structures of all four domains of *C. thermophilum* Mex67–Mtr2 provide atomic resolution information on the Mex67–Mtr2 complex from a single species and indicate that although there is only low sequence identity in some regions within this family of nuclear-export proteins, the folds of the four domains are conserved, demonstrating the highly structurally conserved nature of the NXF family. *C. thermophilum* Mex67–Mtr2 retained features found in both *S. cerevisiae* and *H. sapiens* and conserved the hydrophobic pocket identified as the nucleoporin-binding site found in NTF2L domains from other organisms. The position of the pre-α1 loop, which has been implicated in the spatial arrangement of the Mex67 domains in *S. cerevisiae*, occupied a similar position in *ct*Mex67^NTF2L^ to that observed in *S. cerevisiae* and *H. sapiens*. Notably, Leu368 was positioned in a structurally equivalent position to *sc*Mex67^Leu263^ and *hs*NXF1^Leu370^, indicating that the NTF2L pre-α1 loop position may be conserved across species (Fribourg *et al.*, 2001 ▸; Aibara, Katahira *et al.*, 2015 ▸; Aibara, Valkov *et al.*, 2015 ▸). The homodimeric complex of NTF2L domains in the *ct*Mex67^LRR-NTF2L^ crystals was unanticipated because it had been thought that complex formation between Mex67 and Mtr2 was a prerequisite for efficient nuclear export of mRNA (Santos-Rosa *et al.*, 1998 ▸). Although SEC-MALS indicated that *ct*Mex67^LRR-NTF2L^ is homodimeric in solution, whether Mex67 is ever not complexed to Mtr2 *in vivo* is currently unclear and whether the configuration observed simply represents an inactive form of the Mex67–Mtr2 complex or whether homodimeric Mex67 has another role in the cell has yet to be established.

## Supplementary Material

Supplementary figures. DOI: 10.1107/S2053230X15008766/mn5088sup1.pdf

PDB reference: Mex67 RRM, 4wpm

PDB reference: Mex67 UBA, 4wp2

PDB reference: Mex67 NTF2L, 4wp5

PDB reference: Mex67 LRR, 4wp6

PDB reference: Mtr2, 4x2m

PDB reference: Mex67 LRR+NTF2L, 4xm4

## Figures and Tables

**Table 1:** Data-collection and refinement statistics Values in parentheses are for the outer shell.

	*ct*Mex67^RRM^	*ct*Mex67^LRR^	*ct*Mtr2	*ct*Mex67^NTF2L^Mtr2	*ct*Mex67^LRR-NTF2L^	*ct*Mex67^UBA^
Crystallization condition	2.9*M* ammonium sulfate, 50m*M* TrisHCl pH 8.0	30.9% PEG 4000, 0.15*M* malate, 0.06*M* KSCN	1.26*M* ammonium sulfate, 0.1*M* MES pH 6.0	4*M* NaCl, 0.1*M* MES pH 6.0	16% PEG 3350, 0.1*M* KH_2_PO_4_	1.0*M* sodium citrate, 0.1*M* sodium cacodylate pH 6.9
Data-collection statistics						
Wavelength ()	0.9686	0.9795	0.9686	0.9686	0.9795	0.9200
Space group	*C*2	*C*2	*P*4_1_2_1_2	*P*3_2_21	*P*2_1_2_1_2_1_	*P*3_1_21
Unit-cell parameters						
*a* ()	114.0	114.0	84.0	103.0	43.8	95.9
*b* ()	30.0	33.1	84.0	103.0	96.1	95.9
*c* ()	52.5	43.5	131.3	89.0	195.0	75.0
()	90.0	90.0	90.0	90.0	90.0	90.0
()	96.5	91.7	90.0	90.0	90.0	90.0
()	90.0	90.0	90.0	120.0	90.0	120.0
Resolution range ()	40.72.40 (2.492.40)	43.51.70 (1.731.70)	38.82.00 (2.052.00)	44.72.90 (3.082.90)	48.02.95 (3.052.95)	48.01.70 (1.731.70)
Unique reflections	7066	18096	31939	12265	17888	44143
Total observations	28388	87424	137208	52380	58978	734115
*I*/(*I*)	5.7 (1.8)	9.5 (2.0)	12.2 (1.7)	11.4 (1.3)	11.2 (1.6)	19.8 (2.0)
*R*_merge_ †	0.24 (1.03)	0.11 (0.82)	0.09 (0.84)	0.12 (1.28)	0.11 (0.90)	0.088 (1.56)
*R*_meas_ ‡	0.27 (1.18)	0.124 (0.92)	0.10 (0.95)	0.14 (1.46)	0.13 (1.08)	0.091 (1.62)
*R*_p.i.m._ §	0.14 (0.58)	0.055 (0.42)	0.047 (0.44)	0.067 (0.70)	0.068 (0.58)	0.022 (0.40)
CC_1/2_	0.969 (0.530)	0.996 (0.790)	0.998 (0.622)	0.994 (0.442)	0.996 (0.487)	0.999 (0.717)
Completeness (%)	99.6 (99.7)	99.8 (99.8)	98.7 (98.8)	98.3 (99.7)	99.3 (100)	100 (100)
Multiplicity	4.0	4.8	4.3	4.3	3.4	16.6
Wilson *B* factor (^2^)	24.5	15.4	30.0	76.8	70.3	23.6
Refinement statistics						
Resolution range ()	40.72.40 (2.492.40)	43.51.70 (1.771.70)	38.82.00 (2.062.00)	44.72.90 (3.202.90)	48.02.95 (3.112.95)	41.51.70 (1.731.70)
*R*_work_/*R*_free_ ¶ (%)	22.9/24.6 (32.6/28.1)	18.5/21.3 (25.3/31.0)	18.1/20.2 (26.3/28.1)	22.2/25.4 (34.3/40.9)	22.7/26.5 (30.7/32.1)	19.8/24.0 (29.6/36.5)
Non-H atoms	1401	1308	2863	2964	5274	4034
Ligands	15					72
No. of water molecules	53	86	332	20		359
Bond-length r.m.s.d. ()	0.002	0.008	0.003	0.003	0.003	0.006
Bond-angle r.m.s.d. ()	0.68	1.02	0.79	0.7	0.71	0.98
Ramachandran plot						
Favoured (%)	98.2	98.6	99.1	98.3	98.5	99.1
Outliers (%)	0	0	0	0	0	0
All-atom clashscore††	2.2	2.46	1.6	2.07	2.96	3.19
Average protein *B* factor (^2^)	37.3	25.4	38	83.1	76.0	38.0
Average water *B* factor (^2^)	33.9	33.6	39.3	65.8		39.3
*MolProbity* score (percentile)††	1.09 (100)	1.03 (100)	0.88 (100)	1.41 (100)	1.19 (100)	1.11 (99)

† *R*_merge_ = 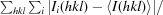 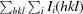, where *I*_*i*_(*hkl*) is an individual intensity measurement and *I*(*hkl*) is the average intensity for all *i* observations of reflection *hkl*.

‡ *R*_meas_ = 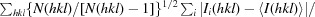 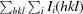, where *I*_*i*_(*hkl*) is an individual intensity measurement and *I*(*hkl*) is the average intensity for all *i* observations of reflection *hkl*.

§ *R*_p.i.m._ = 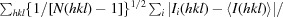 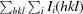, where *I*_*i*_(*hkl*) is an individual intensity measurement and *I*(*hkl*) is the average intensity for all *i* observations of reflection *hkl*.

¶ *R*_work_ = 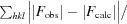 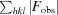, where *F*_obs_ and *F*_calc_ are the observed and calculated structure-factor amplitudes, respectively. *R*_free_ is defined as *R*_work_ for a randomly selected 5% of reflections.

†† The all-atom clashcore is the number of unfavourable all-atom steric overlaps of 0.4 per 1000 atoms (Word *et al.*, 1999 ▸) and the *MolProbity* score (MPscore) is calculated as follows (Keedy *et al.*, 2009 ▸): MPScore = 0.426ln(1 + clashscore) + 0.33ln[1 + max(0, rota_out 1)] + 0.25ln[1 + max(0, rama_iffy 2)] + 0.5.

**Table 2:** Alignments of *C. thermophilum* Mex67Mtr2 and *H. sapiens* NXF1NXT1 The structures of the individual domains from *C. thermophilum* Mex67Mtr2 were compared with the individual domain structures of *H. sapiens* NXF1NXT1 using the *super* command in *PyMOL* using default settings. The global sequence identity between Mex67 and NXF1 was calculated using *NEEDLE*. The sequence identities for the individual domains were calculated by submitting the two PDB files to the *DaliLite* pairwise alignment server. The numbers of residues that were used in the alignment to generate the resulting values are given in parentheses.

	Sequence identity (%)	C r.m.s.d. ()
Global sequence alignment	23	
RRM domain	15 (73)	1.5 (59)
LRR domain	27 (135)	1.5 (116)
NTF2L domain	22 (153)	1.9 (109)
UBA domain	29 (55)	0.75 (41)
Mtr2NXT1	22 (124)	0.70 (75)

**Table 3:** Summary of SAXS data statistics obtained for different constructs of *ct*Mex67Mtr2

Protein sample	Theoretical MW (kDa)	Estimated MW (kDa)	*R*_g_ † (nm)	Real-space *R*_g_ ‡ (nm)	*D*_max_ (nm)
*ct*Mex67^LRR-NTF2L^Mtr2	63.0	64.9	3.01 0.026	3.08 0.012	10.5
*ct*Mex67^UBA^Mtr2	72.5	78.9	3.49 0.027	3.54 0.015	12.2
*ct*Mex67^N^Mtr2	85.5	91.0	3.89 0.033	3.96 0.016	13.6

† Determined by Guinier approximation in *PRIMUS*.

‡ Determined using *GNOM*.
