# Supplementary material for: Structural characterization of the principal mRNA-export factor Mex67–Mtr2 from Chaetomium thermophilum
Source: Acta Crystallogr F Struct Biol Commun. 2015 Jun 27;71(Pt 7):876–88. doi: 10.1107/S2053230X15008766 (PMC4498709; doi:10.1107/S2053230X15008766)
Supplement: Supplementary file 1 [file f-71-00876-sup1.pdf]

Acta Cryst F

STRUCTURAL BIOLOGY COMMUNICATIONS

Volume 71 (2015)

Supporting information for article:

Structural characterization of the principal mRNA export factor Mex67–Mtr2 from *Chaetomium thermophilum*

Shintaro Aibara, Eugene Valkov, Meindert H. Lamers, Lyudmila Dimitrova, Ed Hurt and Murray Stewart

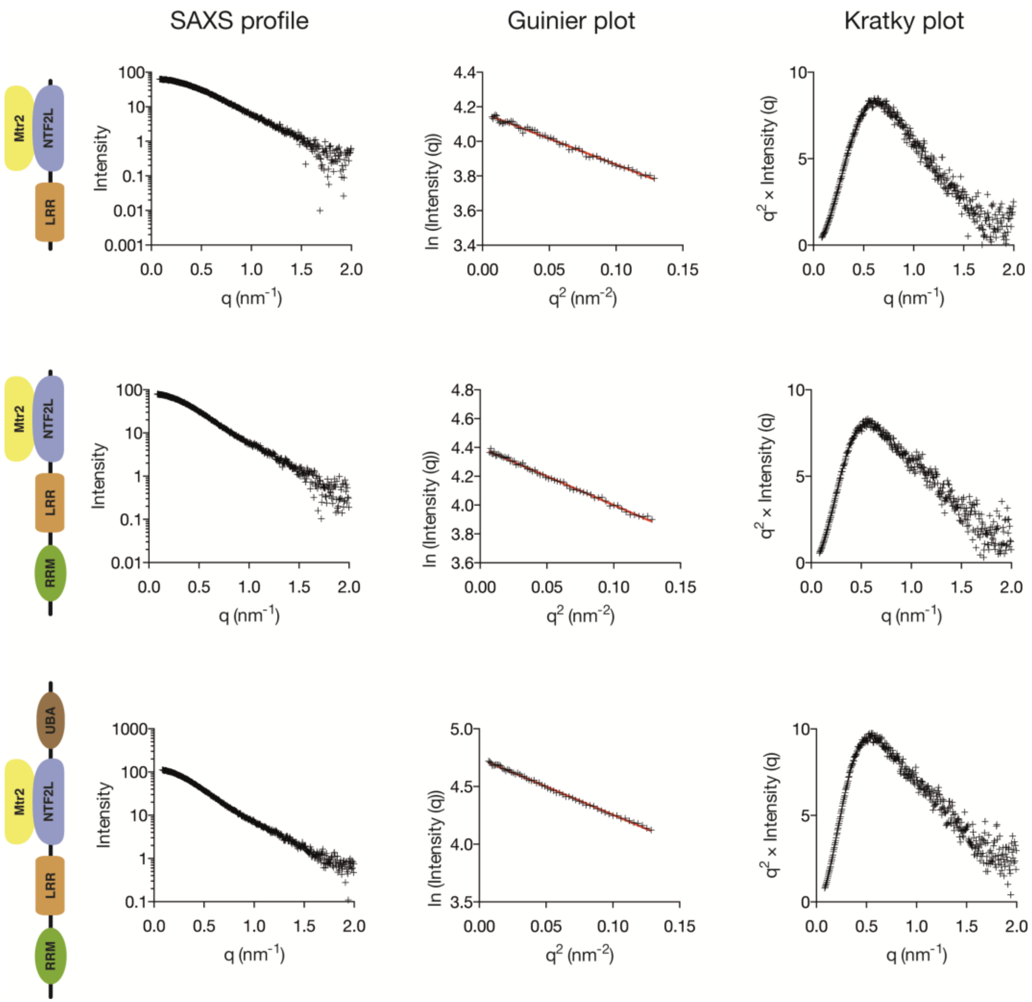

Figure S1 (From left to right) The measured experimental SAXS profile, Guinier plot, and the Kratky plot for (from top to bottom): *ct*Mex67$^{LRR-NTF2L}$:Mtr2, *ct*Mex67$^{\Delta UBA}$:Mtr2, and *ct*Mex67$^{\Delta N}$:Mtr2. Highly schematic representations of the construct used are shown on the left side.

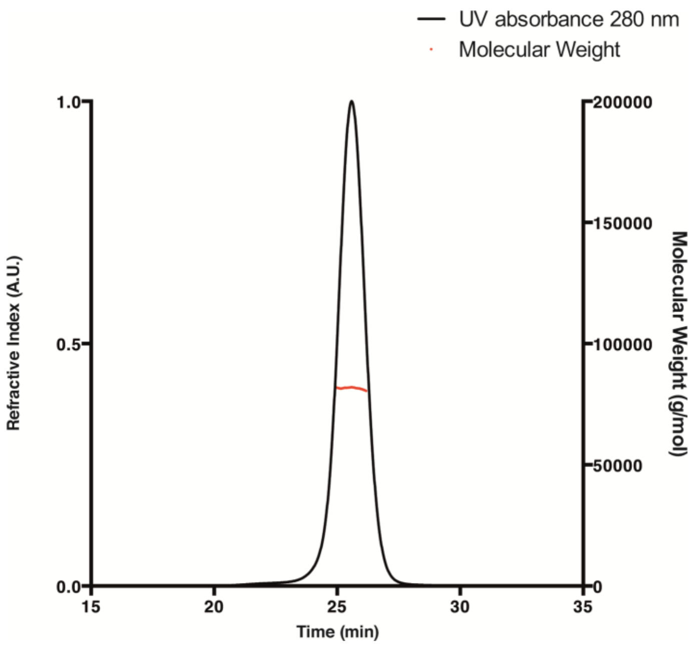

Figure S2 *ct*Mex67$^{LRR\text{-}NTF2L}$ was separated by size-exclusion chromatography and the molecular masses were determined by multiangle light scattering (SEC-MALS). The protein eluted as a single peak with a molecular mass consistent with a dimer in solution.
